# HALP outperforms systemic inflammatory biomarkers for prognosis in locally advanced cervical cancer treated with concurrent chemoradiotherapy

**DOI:** 10.17305/bb.2026.13993

**Published:** 2026-03-10

**Authors:** Xiaojun Zhang, Yilin Yin, Tiantian Yang, Yawen Cong, Shengjun Ji, Yutian Zhao, Yan Kong

**Affiliations:** 1 Department of Radiotherapy and Oncology, Affiliated Hospital of Jiangnan University, Wuxi, Jiangsu, China; 2 Wuxi Medical College, Jiangnan University, Wuxi, Jiangsu, China; 3 Department of Radiotherapy and Oncology, Suzhou Municipal Hospital, The affiliated Suzhou Hospital of Nanjing Medical University, Gusu School, Nanjing Medical University, Suzhou, Jiangsu, China

**Keywords:** Locally advanced cervical cancer, inflammation, biomarker, prognosis

## Abstract

Patients with locally advanced cervical cancer (LACC) treated with concurrent chemoradiotherapy (CCRT) show substantial heterogeneity in survival outcomes, whereas the comparative prognostic value of systemic inflammatory and nutritional biomarkers remains unclear. This study aimed to compare pretreatment inflammatory biomarkers, identify the most informative prognostic indicator, and develop a practical risk-stratification model for LACC. In this multicenter retrospective study, 290 patients with International Federation of Gynecology and Obstetrics (FIGO) stage IIB–IIIC cervical squamous cell carcinoma treated with definitive platinum-based CCRT followed by brachytherapy at two tertiary centers were analyzed. Twelve pretreatment inflammatory and nutritional indices, including the hemoglobin–albumin–lymphocyte–platelet (HALP) score, pan-immune-inflammation value (PIV), and neutrophil-to-lymphocyte ratio (NLR), were evaluated for overall survival (OS) and progression-free survival (PFS) using the concordance index (C-index), time-dependent area under the curve (AUC), and Brier score. Cox regression identified independent prognostic factors, and HALP-based nomograms were internally validated. Among all biomarkers, HALP showed the best and most stable prognostic performance for both OS and PFS, with the highest discrimination and lowest prediction error. Low HALP remained independently associated with worse OS and PFS in multivariable analysis (OS: hazard ratio [HR], 1.654; 95% confidence interval [CI], 1.165–2.366; PFS: HR, 1.702; 95% CI, 1.233–2.344). Nomograms integrating HALP, tumor size, and human papillomavirus (HPV) status showed good calibration and improved predictive accuracy beyond FIGO stage and the baseline clinical model. HALP may therefore serve as a robust, inexpensive, and clinically accessible biomarker for individualized risk stratification in LACC.

## Introduction

Cervical cancer remains one of the most prevalent gynecological malignancies globally and is a leading cause of cancer-related morbidity and mortality among women, particularly in developing regions [1, 2]. While patients diagnosed at early stages often achieve favorable long-term outcomes following standard treatment, a significant proportion present with locally advanced cervical cancer (LACC), for which concurrent chemoradiotherapy (CCRT) is the standard of care [3]. Despite standardized treatment protocols, survival outcomes for LACC patients exhibit substantial variability, with reported recurrence rates ranging from 20% to 40%, alongside marked heterogeneity in progression-free and overall survival (OS) rates [4, 5]. This variability highlights the limited prognostic accuracy of conventional staging systems, such as International Federation of Gynecology and Obstetrics (FIGO) stage, and traditional clinicopathological factors in adequately capturing tumor aggressiveness and individual patient risk.

Emerging evidence suggests that cancer progression and therapeutic response are influenced not only by intrinsic tumor characteristics but also by the host’s systemic inflammatory and immune status [6, 7]. Chronic inflammation promotes tumor development through multiple mechanisms, including cytokine-driven proliferation, angiogenesis, immune evasion, and metastatic spread [8, 9]. Additionally, malnutrition has been associated with immune dysfunction, heightened inflammatory responses, reduced treatment tolerance, and poorer clinical outcomes [10]. Consequently, numerous peripheral blood-derived inflammatory and nutritional indices have been investigated as prognostic biomarkers in cervical cancer due to their simplicity, cost-effectiveness, and clinical feasibility. Among these markers, the neutrophil-to-lymphocyte ratio (NLR) [11, 12], platelet-to-lymphocyte ratio (PLR) [12], lymphocyte-to-monocyte ratio (LMR) [13], systemic immune-inflammation index (SII) [12, 14, 15], systemic inflammation response index (SIRI) [15], pan-immune-inflammation value (PIV) [16], and hemoglobin–albumin–lymphocyte–platelet (HALP) score [17, 18] have demonstrated prognostic significance in LACC patients undergoing radiotherapy or CCRT. However, most previous studies have concentrated on individual biomarkers or a limited set of indices, leading to inconsistent findings and restricting their broader clinical utility. Recent reports indicate that PIV may offer superior prognostic performance compared to conventional inflammatory markers in predicting treatment response and survival outcomes in LACC patients treated with CCRT, underscoring its clinical relevance [16]. Nonetheless, no study to date has comprehensively evaluated and compared an extensive panel of systemic inflammatory biomarkers, including PIV and HALP, within a single cohort of LACC patients.

Thus, the present study aims to conduct a systematic comparative analysis of multiple inflammation-related biomarkers in patients with LACC. This includes PIV, NLR, LMR, PLR, SII, SIRI, neutrophil–albumin ratio (NAR), lymphocyte–albumin ratio (LA), neutrophil–monocyte ratio (NM), neutrophil–platelet ratio (NP), monocyte–platelet ratio (MP), and HALP. By simultaneously assessing these indices, we seek to identify the most robust prognostic marker and develop a more reliable and clinically applicable tool for risk stratification and personalized management of patients with LACC.

## Materials and methods

### Patient selection

This retrospective study consecutively enrolled patients with LACC treated between August 2018 and January 2023 at two tertiary centers: Center 1, The Affiliated Hospital of Jiangnan University, and Center 2, The Affiliated Suzhou Hospital of Nanjing Medical University. LACC was defined as FIGO stage IIB–IIIC, and all patients had histologically confirmed squamous cell carcinoma of the cervix as the sole primary malignancy. Eligible patients received definitive treatment consisting of platinum-based CCRT followed by high-dose-rate intracavitary brachytherapy. Exclusion criteria included the presence of hematologic or autoimmune diseases, distant metastasis (M1), incomplete clinical, laboratory, pathological, or follow-up data, prior anticancer treatment before CCRT, or radical surgery or lymph node staging procedures. At Center 1, 188 patients initially met the inclusion criteria; 56 were excluded, resulting in 132 patients enrolled. At Center 2, 198 patients were screened, 40 were excluded according to the predefined criteria, and 158 patients were ultimately included. In total, 290 patients from both centers were enrolled for further analysis (Figure S1).

A complete-case analysis strategy was employed. Patients were excluded if baseline laboratory parameters required for biomarker calculation, key clinicopathological variables (including tumor size, FIGO stage, or HPV status), or survival follow-up data were missing. Missing data primarily involved baseline hematological measurements and, to a lesser extent, HPV status. Detailed information regarding exclusion reasons is summarized in Table S1. Additionally, the distribution of missing baseline variables by center prior to exclusion is presented in Table S2.

### Treatment and follow-up

All enrolled patients underwent definitive radiotherapy comprising pelvic external beam irradiation combined with intracavitary brachytherapy and concurrent platinum-based chemotherapy. Pelvic external beam radiotherapy was delivered at a total dose of 45–50 Gy, using daily fractions of 1.8–2.0 Gy administered five days per week. For patients with parametrial involvement, a supplemental boost of 5–10 Gy was applied, while those with metastatic pelvic lymph nodes received an additional nodal boost ranging from 10 to 20 Gy. Patients diagnosed with para-aortic lymph node metastasis were treated with extended-field irradiation encompassing the para-aortic region. Intracavitary radiotherapy was performed using a two-dimensional high-dose-rate brachytherapy technique, with treatments delivered once or twice weekly, administering 6–7 Gy per fraction, for a total of 4–5 fractions. The cumulative radiation dose to point A was maintained between 85 and 90 Gy. Concurrent chemotherapy consisted of platinum-based regimens, administered either weekly at a dose of 40 mg/m^2^ or every three weeks at a dose of 75 mg/m^2^, depending on clinical tolerance and institutional practice [19, 20]. Tumor staging was determined per the 2018 International Federation of Gynecology and Obstetrics (FIGO) staging system. Lymph node status was assessed based on imaging evaluation rather than surgical staging. Pelvic and para-aortic lymph node involvement were evaluated using pelvic magnetic resonance imaging and/or contrast-enhanced computed tomography. Lymph nodes were considered positive if they exhibited a short-axis diameter ≥10 mm or displayed suspicious radiologic features, including irregular borders, central necrosis, or heterogeneous enhancement. Pelvic and para-aortic lymph nodes were categorized according to their anatomical location, consistent with the 2018 FIGO staging system.

The survival endpoints evaluated in this study included OS and progression-free survival (PFS). OS was defined as the time from initial diagnosis to death from any cause or the date of last follow-up, while PFS was defined as the time from initial diagnosis to the first documented disease recurrence, disease progression, or death from any cause, whichever occurred first. Disease recurrence or progression was determined based on radiological evidence (computed tomography or magnetic resonance imaging), pathological confirmation when available, or unequivocal clinical progression documented in the medical record. Patients alive without documented recurrence at the last follow-up were censored at the date of last contact. Patients who died without prior documented recurrence were counted as PFS events at the date of death.

Patients were followed through routine clinical and systemic assessments every three months during the first two years following treatment, every six months during years three to five, and annually thereafter, continuing until July 2025. This study was conducted in compliance with the Declaration of Helsinki and was approved by the Ethics Committees of the Affiliated Hospital of Jiangnan University and the Affiliated Suzhou Hospital of Nanjing Medical University.

### Data collection and definition of variables

Clinical data were retrospectively extracted from institutional databases and encompassed demographic, tumor-related, and laboratory information. Baseline characteristics included patient age, while clinicopathological variables comprised histological differentiation, lymph node metastasis status, HPV infection status, primary tumor size, and disease stage as defined by the FIGO classification system. Peripheral blood samples obtained prior to the initiation of CCRT were analyzed to assess pretreatment hematological and biochemical parameters. These measurements included absolute counts of neutrophils, lymphocytes, monocytes, and platelets, as well as hemoglobin concentration and serum albumin levels. Utilizing these routinely available laboratory values, a total of 12 systemic inflammatory and nutritional indices were derived, including the NLR, LMR, PLR, SII, SIRI, NAR, LA, NM, NP, MP, PIV, and HALP score. Detailed formulas for each index are summarized in Table S3.

All laboratory parameters were recorded in the original units reported by the institutional laboratory systems at both participating centers. Hemoglobin and serum albumin were measured in g/L, while absolute counts of neutrophils, lymphocytes, monocytes, and platelets were reported in ×10^9^/L. No unit conversion or rescaling was performed prior to the calculation of inflammatory indices. All biomarker formulas were applied directly using these standardized units to ensure internal consistency across centers.

HPV genotyping was performed at the initial diagnosis before the commencement of CCRT. Cervical exfoliated cells were collected using a cervical brush and preserved in transport medium. HPV DNA detection was conducted using a PCR-based nucleic acid amplification kit (Cape Biochemical Co., Guangzhou, China), followed by hybridization microarray genotyping (HybriMax system). The assay detects 21 HPV subtypes, including 15 high-risk and 6 low-risk types. HPV positivity was defined as the detection of one or more HPV subtypes, while high-risk HPV positivity was defined as the detection of at least one high-risk subtype [19].

### Evaluation metrics for inflammatory biomarkers

To systematically compare the prognostic performance of the 12 systemic inflammatory biomarkers, multiple complementary statistical measures were employed. Discriminative ability was primarily assessed using Harrell’s concordance index (C-index) and time-dependent receiver operating characteristic (ROC) analyses, with the corresponding area under the curve (time-AUC) used to quantify predictive accuracy over time. Higher C-index and time-AUC values indicated better discrimination between patients with differing survival outcomes.

Time-dependent ROC curves and time-AUC values were calculated using inverse probability of censoring weighting to appropriately address right-censored survival data. Model calibration, reflecting the agreement between predicted risks and observed outcomes, was evaluated using the time-dependent Brier score. Brier scores were computed at predefined time points of 12, 36, and 60 months, with lower values indicating superior calibration performance. Bootstrap resampling with 500 iterations was employed to estimate confidence intervals for the C-index, time-AUC, and Brier score, ensuring robustness and stability of performance estimates across biomarkers.

**Figure 1.**
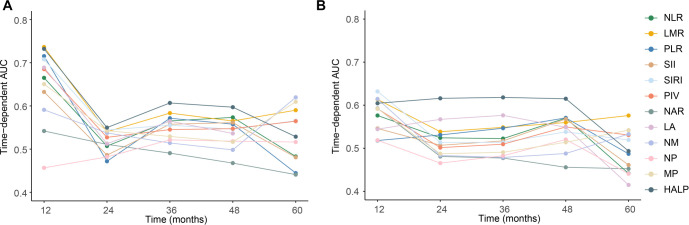
**Time-dependent area under the receiver operating characteristic curves of pretreatment systemic inflammatory and nutritional biomarkers for survival prediction in locally advanced cervical cancer.** (A) Time-dependent area under the receiver operating characteristic curve values for predicting overall survival at 12, 24, 36, 48, and 60 months. The HALP score showed relatively stable and overall superior discriminative performance compared with the other evaluated biomarkers across follow-up. (B) Time-dependent area under the receiver operating characteristic curve values for predicting progression-free survival at 12, 24, 36, 48, and 60 months. The HALP score again demonstrated sustained discrimination over time, whereas most other biomarkers showed lower and less consistent predictive performance. Abbreviations: HALP: Hemoglobin, albumin, lymphocyte, and platelet score; LA: Lymphocyte × albumin; LMR: Lymphocyte-to-monocyte ratio; MP: Monocyte × platelet; NAR: Neutrophil-to-albumin ratio; NLR: Neutrophil-to-lymphocyte ratio; NM: Neutrophil × monocyte; NP: Neutrophil × platelet; PIV: Pan-immune-inflammation value; PLR: Platelet-to-lymphocyte ratio; SII: Systemic immune-inflammation index; SIRI: Systemic inflammation response index.

The “Time-AUC” reported in Table 1 represents the integrated time-dependent AUC, calculated as the weighted average of AUC(t) over the evaluation window from 12–60 months using inverse probability of censoring weighting. Similarly, the “Time-Brier Score” corresponds to the integrated Brier score over the same time interval. In contrast, Figure 1 presents landmark AUC values at specific time points (12, 24, 36, 48, and 60 months).

**Table 1:** Comparative analysis of twelve inflammatory biomarkers in predicting overall survival and progression-free survival

**Biomarkers**	**Overall survival**			**Progression-free survival**		
	**C-index (95% CI)**	**Time-AUC (95% CI)**	**Time-Brier score (95% CI)**	**C-index** **(95% CI)**	**Time-AUC (95% CI)**	**Time-Brier score (95% CI)**
NLR	0.534 (0.479, 0.589)	0.559 (0.450, 0.668)	0.172 (0.159, 0.182)	0.538 (0.492, 0.585)	0.527 (0.426, 0.627)	0.198 (0.186, 0.206)
LMR	0.565 (0.508, 0.621)	0.601 (0.490, 0.716)	0.171 (0.155, 0.181)	0.561 (0.515, 0.608)	0.567 (0.469, 0.666)	0.197 (0.184, 0.207)
PLR	0.541 (0.486, 0.596)	0.552 (0.445, 0.659)	0.172 (0.155, 0.183)	0.549 (0.504, 0.595)	0.531 (0.432, 0.629)	0.198 (0.182, 0.208)
SII	0.538 (0.485, 0.591)	0.544 (0.436, 0.651)	0.172 (0.160, 0.182)	0.536 (0.489, 0.583)	0.520 (0.420, 0.619)	0.198 (0.186, 0.208)
SIRI	0.552 (0.497, 0.608)	0.584 (0.476, 0.692)	0.171 (0.158, 0.180)	0.546 (0.499, 0.593)	0.543 (0.446, 0.640)	0.197 (0.186, 0.207)
PIV	0.554 (0.499, 0.610)	0.574 (0.468, 0.679)	0.172 (0.157, 0.182)	0.543 (0.496, 0.590)	0.537 (0.436, 0.637)	0.198 (0.185, 0.207)
NAR	0.505 (0.451, 0.559)	0.490 (0.384, 0.597)	0.172 (0.158, 0.182)	0.506 (0.457, 0.554)	0.468 (0.372, 0.564)	0.197 (0.183, 0.207)
LA	0.524 (0.470, 0.578)	0.538 (0.431, 0.645)	0.172 (0.157, 0.183)	0.553 (0.507, 0.600)	0.530 (0.437, 0.624)	0.198 (0.185, 0.206)
NM	0.536 (0.482, 0.591)	0.552 (0.450, 0.654)	0.171 (0.154, 0.182)	0.519 (0.471, 0.568)	0.519 (0.419, 0.620)	0.198 (0.184, 0.207)
NP	0.526 (0.471, 0.580)	0.499 (0.383, 0.615)	0.172 (0.158, 0.183)	0.507 (0.456, 0.557)	0.486 (0.386, 0.585)	0.198 (0.183, 0.209)
MP	0.562 (0.509, 0.616)	0.570 (0.469, 0.670)	0.172 (0.158, 0.184)	0.534 (0.484, 0.584)	0.525 (0.422, 0.629)	0.198 (0.183, 0.207)
HALP	0.609 (0.554, 0.663)	0.603 (0.492, 0.714)	0.170 (0.154, 0.183)	0.605 (0.560, 0.650)	0.609 (0.509, 0.709)	0.195 (0.180, 0.206)

Abbreviations: C-index: Concordance index; CI: Confidence interval; LA: Lymphocyte × albumin; LMR: Lymphocyte-to-monocyte ratio; MP: Monocyte × platelet; NAR: Neutrophil-to-albumin ratio; NLR: Neutrophil-to-lymphocyte ratio; NM: Neutrophil × monocyte; NP: Neutrophil × platelet; PIV: Pan-immune-inflammation value; PLR: Platelet-to-lymphocyte ratio; SII: Systemic immune-inflammation index; SIRI: Systemic inflammation response index; Time-AUC: Time-dependent area under the receiver operating characteristic curve; Time-Brier Score: Time-dependent Brier score.

**Table 2:** Baseline clinicopathological characteristics of patients with locally advanced cervical cancer in this study

**Variables**	**All patients (*n* ═ 290)**
Age (year, %)	
< 60	196 (67.59)
≥ 60	94 (32.41)
Differentiation (%)	
Well	28 (9.66)
Moderate	208 (71.72)
Poor	54 (18.62)
Tumor size (%)	
< 4 cm	67 (23.10)
≥ 4 cm	223 (76.90)
FIGO (%)	
IIB	96 (33.10)
IIIA	28 (9.66)
IIIB	80 (27.59)
IIIC	86 (29.66)
Lymph node metastasis	
Negative	204 (70.34)
PLN only	61 (21.03)
PLN and PALN	25 (8.62)
HPV infection (%)	
Negative	145 (50.00)
Positive	145 (50.00)
NLR (median [IQR])	2.11 (1.65, 3.03)
LMR (median [IQR])	3.63 (2.84, 5.11)
PLR (median [IQR])	136.76 (101.54, 192.18)
SII (median [IQR])	558.02 (365.93, 811.26)
SIRI (median [IQR])	1.12 (0.71, 1.70)
PIV (median [IQR])	285.52 (157.53, 477.58)
NAR (median [IQR])	1.01 (0.79, 1.25)
LA (median [IQR])	7.68 (5.92, 9.78)
NM (median [IQR])	2.04 (1.31, 3.11)
NP (median [IQR])	1044.01 (746.24, 1432.26)
MP (median [IQR])	131.92 (95.99, 166.50)
HALP (median [IQR])	38.96 (26.12, 50.20)

Abbreviations: FIGO: International Federation of Gynecology and Obstetrics; HALP: Hemoglobin, albumin, lymphocyte, and platelet score; HPV: Human papillomavirus; IQR: Interquartile range; LA: Lymphocyte × albumin; LMR: Lymphocyte-to-monocyte ratio; MP: Monocyte × platelet; NAR: Neutrophil-to-albumin ratio; NLR: Neutrophil-to-lymphocyte ratio; NM: Neutrophil × monocyte; NP: Neutrophil × platelet; PIV: Pan-immune-inflammation value; PALN: Para-aortic lymph node; PLN: Pelvic lymph node; PLR: Platelet-to-lymphocyte ratio; SII: Systemic immune-inflammation index; SIRI: Systemic inflammation response index.

### Development and assessment of nomogram

Potential prognostic factors for OS and PFS were initially explored using univariate Cox proportional hazards models. Variables demonstrating significant associations were subsequently entered into a multivariate Cox regression with a stepwise selection procedure to identify independent predictors. Based on these results, a baseline clinical model was constructed using the identified independent variables, with the HALP index intentionally excluded to serve as a reference framework.

Nomogram models were developed by incorporating HALP alongside the remaining independent prognostic factors to generate individualized predictions of OS and PFS. The predictive performance of the nomograms was evaluated through time-dependent ROC analyses and calibration plots, which assessed discrimination and agreement between predicted and observed outcomes, respectively. The additional prognostic contribution of the nomograms relative to the baseline clinical model and the FIGO staging system was quantified using net reclassification improvement (NRI) and integrated discrimination improvement (IDI) metrics, with confidence intervals estimated via bootstrap resampling.

Internal validation and correction for potential overfitting were conducted using bootstrap resampling with 500 iterations. Within each bootstrap sample, the nomogram was refitted using the same variable selection strategy, and model performance—including the C-index, time-dependent AUC, Brier score, and calibration—was assessed in both the resampled and original datasets. Optimism was calculated as the average difference between apparent and test performance across resamples, and optimism-adjusted estimates were obtained by subtracting this value from the apparent performance measures.

### Statistical analysis

The distribution of continuous variables was evaluated using the Kolmogorov–Smirnov test. Normally distributed data are presented as mean ± standard deviation, whereas non-normally distributed variables are summarized as median values with interquartile ranges (IQR). Group comparisons for continuous variables were conducted using the Student’s *t*-test or the Mann–Whitney *U* test, depending on data distribution, while categorical variables were compared using the chi-square test or Fisher’s exact test, as appropriate. Associations between potential prognostic factors and survival outcomes were first explored using univariate Cox proportional hazards regression. Variables demonstrating statistical significance were subsequently entered into a multivariate Cox regression model with inverse stepwise selection to identify independent predictors. The proportional hazards assumption for the HALP index in both OS and PFS models was assessed using Schoenfeld residuals. To investigate potential non-linear relationships between HALP and survival outcomes, restricted cubic spline functions with three knots were incorporated into Cox proportional hazards models. Wald chi-square tests were applied to evaluate both the overall effect and the presence of nonlinear components. All statistical analyses were performed using R software (version 4.2.1), and statistical significance was defined as a two-sided *P* value of less than 0.05.

### Ethical statement

The studies involving human participants were reviewed and approved by the Ethics Committee of The Affiliated Hospital of Jiangnan University and The Affiliated Suzhou Hospital of Nanjing Medical University. The patients/participants provided their written informed consent to participate in this study.

## Results

### Patient characteristics

This study included a total of 290 patients diagnosed with LACC. The median age of the cohort was 56 years (IQR, 49–63 years). A majority of the patients, 196 (67.6%), were younger than 60 years, while 94 patients (32.4%) were aged 60 years or older. Regarding tumor differentiation, most patients exhibited moderately differentiated tumors (71.7%), with well-differentiated and poorly differentiated tumors accounting for 9.7% and 18.6% of cases, respectively. The study population predominantly presented with large primary tumors; 223 patients (76.9%) had tumors measuring ≥ 4 cm in diameter, while only 67 patients (23.1%) had tumors < 4 cm. According to the FIGO staging system, 96 patients (33.1%) were classified as stage IIB, 28 (9.7%) as stage IIIA, 80 (27.6%) as stage IIIB, and 86 (29.7%) as stage IIIC, indicating that nearly two-thirds of patients were diagnosed with stage III disease. Lymph node metastasis was absent in 204 patients (70.3%); 61 patients (21.0%) exhibited pelvic lymph node involvement only, and 25 patients (8.6%) had both pelvic and para-aortic lymph node metastases. HPV infection status was evenly distributed, with 145 patients (50.0%) testing positive and 145 (50.0%) testing negative. Baseline systemic inflammatory and nutritional indices exhibited substantial interindividual variability. The median NLR was 2.11 (IQR, 1.65–3.03), and the median LMR was 3.63 (IQR, 2.84–5.11). The PLR showed a median value of 136.76 (IQR, 101.54–192.18). Composite inflammatory indices revealed median values of 558.02 (IQR, 365.93–811.26) for the SII, 1.12 (IQR, 0.71–1.70) for the SIRI, and 285.52 (IQR, 157.53–477.58) for the PIV. Nutritional and mixed indices, including the NAR, LA, NM, NP, MP, and HALP score, also demonstrated wide distributions, with a median HALP score of 38.96 (IQR, 26.12–50.20). These baseline characteristics are summarized in Table 2. The median follow-up duration was 58 months, ranging from 31 to 78 months. During the follow-up period, 132 deaths were recorded for OS. For PFS, 159 events, including recurrence and death, were observed.

**Table 3:** Association of HALP with clinicopathological characteristics in patients with locally advanced cervical cancer

**Variables**	**High HALP (*n* ═ 145)**	**Low HALP (*n* ═ 145)**	***P* value**
Age (year, %)			0.171
< 60	104 (71.72)	92 (63.45)	
≥ 60	41 (28.28)	53 (36.55)	
Differentiation (%)			0.724
Well	16 (11.03)	12 (8.28)	
Moderate	103 (71.03)	105 (72.41)	
Poor	26 (17.93)	28 (19.31)	
Tumor size (%)			0.782
< 4 cm	35 (24.14)	32 (22.07)	
≥ 4 cm	110 (75.86)	113 (77.93)	
FIGO (%)			0.563
IIB	47 (32.41)	49 (33.79)	
IIIA	12 (8.28)	16 (11.03)	
IIIB	45 (31.03)	35 (24.14)	
IIIC	41 (28.28)	45 (31.03)	
Lymph node metastasis (%)			0.802
Negative	104 (71.72)	100 (68.97)	
PLN only	30 (20.69)	31 (21.38)	
PLN and PALN	11 (7.59)	14 (9.66)	
HPV infection (%)			0.102
Negative	80 (55.17)	65 (44.83)	
Positive	65 (44.83)	80 (55.17)	
NLR (median [IQR])	1.81 (1.38, 2.30)	2.60 (2.07, 3.65)	<0.001
LMR (median [IQR])	4.34 (3.42, 5.76)	3.11 (2.36, 3.98)	<0.001
PLR (median [IQR])	110.98 (83.84, 134.01)	173.68 (139.67, 239.74)	<0.001
SII (median [IQR])	428.80 (293.06, 572.60)	740.33 (510.69, 1065.06)	<0.001
SIRI (median [IQR])	0.85 (0.57, 1.38)	1.36 (0.87, 2.26)	<0.001
PIV (median [IQR])	196.85 (126.00, 347.59)	393.04 (238.47, 645.13)	<0.001
NAR (median [IQR])	1.00 (0.71, 1.18)	1.04 (0.85, 1.37)	0.003
LA (median [IQR])	9.24 (7.39, 10.77)	6.72 (4.62, 8.36)	<0.001
NM (median [IQR])	1.94 (1.14, 2.69)	2.16 (1.46, 3.36)	0.002
NP (median [IQR])	942.76 (669.78, 1316.72)	1215.50 (785.84, 1824.34)	<0.001
MP (median [IQR])	113.96 (77.70, 149.38)	144.16 (113.24, 202.37)	<0.001
HALP (median [IQR])	50.22 (43.54, 60.36)	26.03 (21.04, 31.75)	<0.001

Abbreviations: FIGO: International Federation of Gynecology and Obstetrics; HALP: Hemoglobin, albumin, lymphocyte, and platelet score; HPV: Human papillomavirus; IQR: Interquartile range; LA: Lymphocyte × albumin; LMR: Lymphocyte-to-monocyte ratio; MP: Monocyte × platelet; NAR: Neutrophil-to-albumin ratio; NLR: Neutrophil-to-lymphocyte ratio; NM: Neutrophil × monocyte; NP: Neutrophil × platelet; PIV: Pan-immune-inflammation value; PLAN: Para-aortic lymph node; PLN: Pelvic lymph node; PLR: Platelet-to-lymphocyte ratio; SII: Systemic immune-inflammation index; SIRI: Systemic inflammation response index.

### Optimal survival prediction inflammation biomarkers

The prognostic performance of 12 systemic inflammatory biomarkers for OS and PFS was thoroughly evaluated using discrimination and calibration metrics, including the C-index, time-dependent AUC, and time-dependent Brier score (Table 1). Notably, substantial heterogeneity was observed among the biomarkers concerning their predictive accuracy, with the HALP score consistently demonstrating superior performance across multiple evaluation indices.

Among all evaluated biomarkers, HALP achieved the highest discriminative ability for both OS and PFS. Specifically, HALP yielded a C-index of 0.609 (95% CI, 0.554–0.663) for OS and 0.605 (95% CI, 0.560–0.650) for PFS, outperforming traditional inflammatory markers such as NLR, LMR, PLR, SII, SIRI, and PIV. Furthermore, HALP exhibited the highest time-dependent AUC values for OS (0.603, 95% CI, 0.492–0.714) and PFS (0.609, 95% CI, 0.509–0.709), indicating its superior capacity to distinguish patients with varying survival outcomes over time. Consistently, HALP also recorded the lowest time-dependent Brier scores among all biomarkers for both OS and PFS, reflecting improved calibration and reduced prediction error. The dynamic predictive performance of each inflammatory biomarker is illustrated in Figure 1. For OS prediction (Figure 1A), HALP maintained relatively stable and consistently higher time-AUC values across the 12-, 24-, 36-, 48-, and 60-month time points compared to other indices, whereas most conventional markers exhibited marked fluctuations and a decline in predictive accuracy over longer follow-up periods. A similar pattern was observed for PFS (Figure 1B), where HALP again demonstrated sustained discrimination across all evaluated time horizons, while other biomarkers exhibited modest and less consistent performance. Although several biomarkers, including LMR, MP, and PIV, displayed moderate prognostic ability, their C-index and time-AUC values were inferior to those of HALP. In contrast, markers such as NAR and NP demonstrated limited discriminative power, with C-index values approximating 0.50 for both OS and PFS, suggesting minimal prognostic utility. Collectively, these inflammatory indices exhibited comparable time-dependent Brier scores; however, HALP consistently achieved the lowest values, further supporting its superior overall predictive performance.

### Characteristics and survival analysis of HALP

Patients were stratified into low HALP (< 38.96) and high HALP (≥ 38.96) groups based on the cohort-specific median HALP value. This median split was intentionally adopted to avoid outcome-driven threshold optimization and to ensure methodological consistency across the comparative biomarker analysis. HALP was also evaluated as a continuous variable in Cox regression and restricted cubic spline models to preserve prognostic information and assess potential nonlinearity. Comparisons of baseline clinicopathological characteristics revealed no significant differences between the two groups concerning age, tumor differentiation, tumor size, FIGO stage, lymph node metastasis status, or HPV infection status (all *P* > 0.05; Table 3). These findings indicate that HALP stratification was largely independent of conventional clinicopathological factors. In contrast, significant differences were observed in systemic inflammatory and nutritional biomarkers between the two groups. Patients in the high-HALP group exhibited significantly lower levels of NLR, PLR, SII, SIRI, PIV, NAR, NM, NP, and MP, alongside higher LMR and LA values compared to those in the low-HALP group (all *P* < 0.01). As anticipated, the median HALP value was substantially higher in the high-HALP group (50.22, IQR, 43.54–60.36) than in the low-HALP group (26.03, IQR, 21.04–31.75; *P* < 0.001). Collectively, these results suggest that a higher HALP score reflects a more favorable immune-inflammatory and nutritional status.

**Figure 2.**
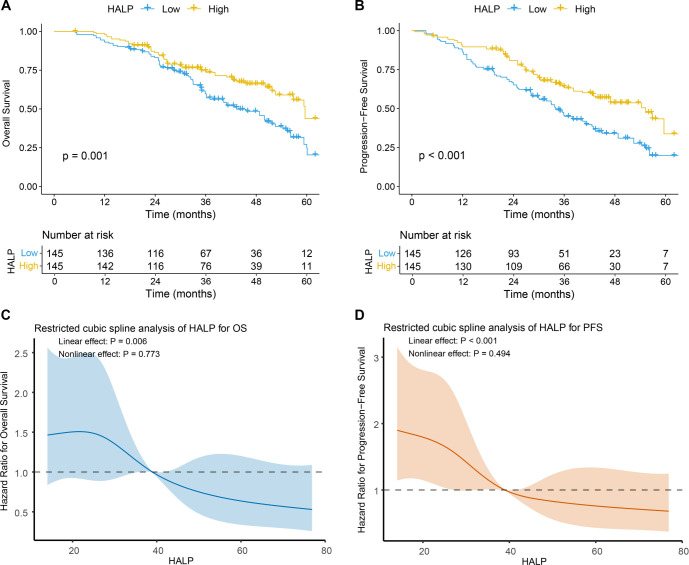
**Kaplan–Meier survival curves and restricted cubic spline analyses of the HALP score in patients with locally advanced cervical cancer.** (A) Kaplan–Meier curves for overall survival according to low and high HALP groups. Patients with high HALP had significantly better overall survival than those with low HALP (log-rank *P* ═ 0.001), with persistent separation of the survival curves throughout follow-up. Numbers at risk are shown below the plot. (B) Kaplan–Meier curves for progression-free survival according to low and high HALP groups. Patients with high HALP had significantly better progression-free survival than those with low HALP (log-rank *P* < 0.001), and the survival difference was maintained over time. Numbers at risk are shown below the plot. (C) Restricted cubic spline analysis of HALP as a continuous variable for overall survival. Increasing HALP was associated with a progressively lower risk of death in a significant linear manner (*P* ═ 0.006), with no evidence of a nonlinear association (*P* ═ 0.773). The solid line represents the estimated effect, and the shaded area indicates the 95% confidence interval. (D) Restricted cubic spline analysis of HALP as a continuous variable for progression-free survival. Increasing HALP was associated with a progressively lower risk of disease progression or death in a significant linear manner (*P* < 0.001), with no evidence of a nonlinear association (*P* ═ 0.494). The solid line represents the estimated effect, and the shaded area indicates the 95% confidence interval. Abbreviations: HALP: Hemoglobin–albumin–lymphocyte–platelet score; OS: Overall survival; PFS: Progression-free survival.

Kaplan–Meier survival analyses demonstrated that patients with high HALP had significantly better survival outcomes than those with low HALP. The high-HALP group exhibited superior OS (log-rank *P* ═ 0.001; Figure 2A) and PFS (log-rank *P* < 0.001; Figure 2B). Throughout the follow-up period, the separation between the survival curves was consistently maintained, indicating a durable prognostic effect of HALP over time. To further characterize the relationship between HALP and survival outcomes, restricted cubic spline analyses were performed treating HALP as a continuous variable. For OS, a significant linear association between HALP and mortality risk was observed (*P* ═ 0.006), while no evidence of a nonlinear relationship was detected (*P* ═ 0.773; Figure 2C). Similarly, HALP demonstrated a significant linear association with PFS (*P* < 0.001), with no significant nonlinear component (*P* ═ 0.494; Figure 2D). Increasing HALP values were associated with a progressively reduced hazard of death and disease progression.

Univariate and multivariate Cox proportional hazards analyses were conducted to identify factors associated with OS and PFS in patients with LACC (Table 4). In univariate analyses, a low HALP score was significantly correlated with inferior OS (HR = 1.772, 95% CI: 1.243–2.524, *P* ═ 0.001) and PFS (HR = 1.794, 95% CI: 1.301–2.472, *P* < 0.001). After adjustment for potential confounders in multivariate models, HALP remained an independent prognostic factor for both OS (HR = 1.654, 95% CI: 1.165–2.366, *P* ═ 0.005) and PFS (HR = 1.702, 95% CI: 1.233–2.344, *P* ═ 0.001), indicating that patients with low HALP faced a significantly higher risk of death and disease progression compared to those with high HALP. Tumor size also demonstrated a significant prognostic impact. Patients with tumors ≥ 4 cm exhibited worse OS (univariate HR = 1.790, *P* ═ 0.016) and PFS (univariate HR = 1.642, *P* ═ 0.019), and tumor size remained independently associated with both OS (multivariate HR = 1.694, 95% CI: 1.053–2.732, *P* ═ 0.031) and PFS (multivariate HR = 1.593, 95% CI: 1.051–2.402, *P* ═ 0.029). Furthermore, HPV infection status was significantly correlated with survival outcomes. HPV-positive patients had poorer OS (univariate HR = 1.582, *P* ═ 0.009) and PFS (univariate HR = 1.733, *P* < 0.001), and HPV infection remained an independent adverse prognostic factor in multivariate analyses for both OS (HR = 1.463, 95% CI: 1.033–2.074, *P* ═ 0.032) and PFS (HR = 1.671, 95% CI: 1.213–2.314, *P* ═ 0.001). The proportional hazards assumption for HALP was not violated in either the OS or PFS models, as evidenced by Schoenfeld residual analyses showing no systematic time-dependent trends (Figure S2).

**Table 4:** Univariate and multivariate analyses of prognostic factors in locally advanced cervical cancer

**Variables**	**Overall survival**				**Progression free survival**			
	**Univariate analysis**		**Multivariate analysis**		**Univariate analysis**		**Multivariate analysis**	
	**HR (95% CI)**	***P* value**	**HR (95% CI)**	***P* value**	**HR (95% CI)**	***P* value**	**HR (95% CI)**	***P* value**
HALP (low vs. high)	1.772 (1.243–2.524)	0.001	1.654 (1.165–2.366)	0.005	1.794 (1.301–2.472)	<0.001	1.702 (1.233–2.344)	0.001
Age (≥ 60 year vs. < 60 year)	0.769 (0.534–1.122)	0.168			0.907 (0.651–1.262)	0.566		
Differentiation (poor vs. moderate)	0.944 (0.583–1.534)	0.815			0.949 (0.623–1.442)	0.806		
Differentiation (well vs. moderate)	1.26 (0.733–2.182)	0.401			1.101 (0.652–1.864)	0.718		
FIGO (III vs. II)	1.25 (0.872–1.814)	0.224			1.222 (0.874–1.702)	0.244		
Tumor size (≥ 4 cm vs. < 4 cm)	1.79 (1.112–2.893)	0.016	1.694 (1.053–2.732)	0.031	1.642 (1.084–2.482)	0.019	1.593 (1.051–2.402)	0.029
Lymph node metastasis (PLN and PALN vs. negative)	1.44 (0.803–2.582)	0.222			1.431 (0.833–2.472)	0.193		
Lymph node metastasis (PLN only vs. negative)	1.154 (0.749–1.772)	0.519			1.142 (0.773–1.672)	0.515		
HPV infection (positive vs. negative)	1.582 (1.121–2.233)	0.009	1.463 (1.033–2.074)	0.032	1.733 (1.262–2.381)	<0.001	1.671 (1.213–2.314)	0.001

Abbreviations: CI: Confidence interval; FIGO: International Federation of Gynecology and Obstetrics; HALP: Hemoglobin, albumin, lymphocyte, and platelet score; HPV: Human papillomavirus; HR: Hazard ratio; PLAN: Para-aortic lymph node; PLN: Pelvic lymph node.

### Development and assessment of nomograms

**Figure 3.**
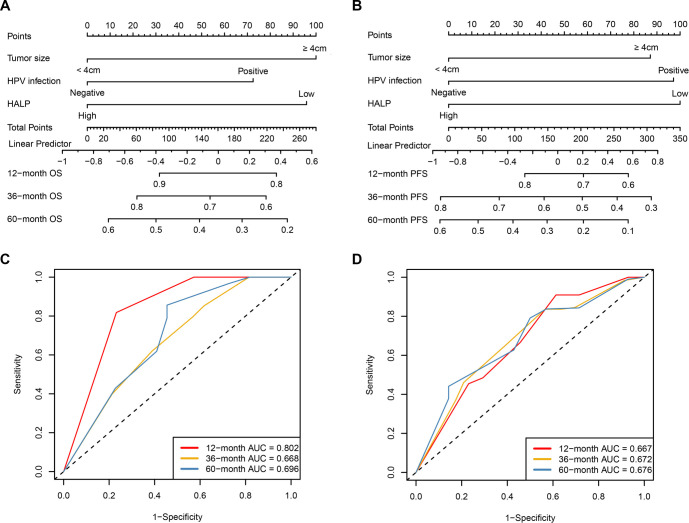
**Nomograms for individualized prediction of overall survival and progression-free survival and their time-dependent ROC performance.** (A) Nomogram for predicting 12-, 36-, and 60-month OS based on tumor size, HPV infection status, and HALP. Each variable is assigned a weighted score, and the total score corresponds to the linear predictor and estimated probability of OS. (B) Nomogram for predicting 12-, 36-, and 60-month PFS based on tumor size, HPV infection status, and HALP. The total score is translated into individualized probabilities of PFS, with higher scores indicating a less favorable prognosis. (C) Time-dependent ROC curves of the OS nomogram at 12, 36, and 60 months, with corresponding AUC values of 0.802, 0.668, and 0.696, respectively. (D) Time-dependent ROC curves of the PFS nomogram at 12, 36, and 60 months, with corresponding AUC values of 0.667, 0.672, and 0.676, respectively. Abbreviations: AUC: Area under the curve; HALP: Hemoglobin, albumin, lymphocyte, and platelet score; HPV: Human papillomavirus; OS: Overall survival; PFS: Progression-free survival; ROC: Receiver operating characteristic.

**Figure 4.**
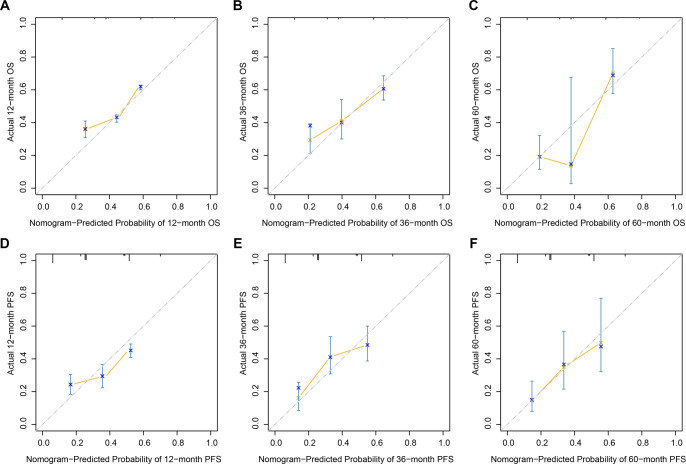
**Calibration plots of the nomograms for predicting overall survival and progression-free survival.** Calibration plots showing the agreement between nomogram-predicted and observed probabilities of 12-month (A), 36-month (B), and 60-month (C) OS, and 12-month (D), 36-month (E), and 60-month (F) PFS in patients with locally advanced cervical cancer, demonstrating good calibration across the evaluated time points. Abbreviations: OS: Overall survival; PFS: Progression-free survival.

Based on the findings from multivariate Cox regression analyses, nomogram models were developed to offer individualized predictions of OS and PFS in patients with LACC (Figure 3A–B). The independent prognostic variables integrated into both nomograms included tumor size, HPV infection status, and HALP, with each factor assigned a weighted score corresponding to its contribution to survival risk. By summing these individual scores, a total point value for each patient was calculated, which could then be translated into estimated probabilities of 12-, 36-, and 60-month OS and PFS. The discriminatory ability of the nomograms was assessed using time-dependent ROC analyses (Figure 3C–D). For OS prediction, the nomogram achieved AUC values of 0.802, 0.668, and 0.696 at 12, 36, and 60 months, respectively, indicating strong short-term and acceptable long-term predictive accuracy. Similarly, the PFS nomogram exhibited AUC values of 0.667, 0.672, and 0.676 at the same time points, demonstrating stable discrimination across various follow-up intervals. Calibration performance was further evaluated by comparing nomogram-predicted survival probabilities with observed outcomes at 12, 36, and 60 months (Figure 4A–F). Calibration plots for both OS and PFS indicated strong agreement between predicted and actual survival probabilities.

To quantify the incremental predictive value of the nomogram relative to the baseline clinical model and the FIGO staging system, time-to-event NRI and IDI were calculated at the 60-month time horizon using a continuous (category-free) approach. Censoring was addressed using inverse probability of censoring weighting (IPCW), and confidence intervals were determined through bootstrap resampling with 500 iterations.

### Model comparison

The predictive performance of the nomogram was further compared with the FIGO staging system and the baseline clinical model using the C-index, IDI, and NRI (Table 5). For OS, the nomogram demonstrated the highest discriminatory ability, achieving a C-index of 0.708 (95% CI: 0.664–0.751), surpassing both the clinical model (C-index: 0.643, 95% CI: 0.595–0.691) and the FIGO staging system (C-index: 0.607, 95% CI: 0.528–0.686). Relative to the clinical model, the nomogram provided a significant improvement in discrimination, evidenced by an IDI of 0.068 (95% CI: 0.014–0.124, *P* ═ 0.003) and an NRI of 0.214 (95% CI: 0.132–0.296, *P* < 0.001). Conversely, the FIGO system exhibited inferior predictive performance, with negative IDI and NRI values compared to the clinical model, indicating suboptimal risk reclassification. Similar findings were observed for PFS, where the nomogram achieved a C-index of 0.695 (95% CI: 0.632–0.748), outpacing both the clinical model (C-index: 0.664, 95% CI: 0.541–0.694) and FIGO staging (C-index: 0.619, 95% CI: 0.518–0.720). The incorporation of the nomogram resulted in a significant enhancement in predictive accuracy, with an IDI of 0.079 (95% CI: 0.018–0.135, *P* ═ 0.002) and an NRI of 0.226 (95% CI: 0.121–0.331, *P* < 0.001) relative to the clinical model.

**Table 5:** Comparative analysis of the predictive value of the nomogram, FIGO system, and clinical model

**Models**	**C-index**		**IDI**		**NRI**	
	**Value**	**95% CI**	**Difference**	***P* value**	**Difference**	***P* value**
OS						
Nomogram	0.708	(0.664, 0.751)	0.068 (0.014, 0.124)	0.003	0.214 (0.132, 0.296)	<0.001
FIGO	0.607	(0.528, 0.686)	--0.051 (--0.112, --0.006)	<0.001	--0.158 (--0.214, --0.101)	<0.001
Clinical model	0.643	(0.595, 0.691)	Ref.		Ref.	
PFS						
Nomogram	0.695	(0.632, 0.748)	0.079 (0.018, 0.135)	0.002	0.226 (0.121, 0.331)	<0.001
FIGO	0.619	(0.518, 0.720)	--0.058 (--0.119, --0.007)	<0.001	--0.169 (--0.247, --0.129)	<0.001
Clinical model	0.664	(0.541, 0.694)	Ref.		Ref.	

Note: NRI and IDI were calculated at the 60-month horizon using a continuous (category-free) approach, incorporating inverse probability of censoring weighting to address right-censored survival data. Confidence intervals were estimated through bootstrap resampling with 500 iterations. Abbreviations: C-index: Concordance index; CI: Confidence interval; FIGO: International Federation of Gynecology and Obstetrics; IDI: Integrated discrimination improvement; NRI: Net reclassification improvement; OS: Overall survival; PFS: Progression-free survival.

## Discussion

In this multicenter retrospective study, we conducted a comprehensive comparison of 12 systemic inflammatory and nutritional biomarkers in patients with LACC undergoing definitive CCRT. Our findings indicate that the HALP score provides the most robust and consistent prognostic value for both OS and PFS. Among all evaluated indices, HALP exhibited superior discrimination, stable time-dependent predictive performance, and the lowest prediction error, outperforming widely used markers such as NLR, PLR, SII, SIRI, and PIV. Notably, HALP remained an independent prognostic factor even after adjustment for established clinicopathological variables, with its prognostic effect being linear and sustained over long-term follow-up. By integrating HALP with tumor size and HPV status, we developed nomogram models that significantly enhanced individualized risk stratification compared with the FIGO staging system and a baseline clinical model. These findings underscore the clinical relevance of incorporating systemic host factors into prognostic assessments for patients with LACC. In modern radiation oncology, predictive modeling has increasingly expanded beyond tumor control probability to encompass normal tissue complication probability (NTCP) and individualized risk estimation, reflecting a broader trend towards quantitative, model-based treatment planning [21]. Integrating biomarkers such as HALP into prognostic frameworks may further refine individualized therapeutic decision-making.

The consistent prognostic performance of HALP observed in our cohort is biologically plausible and supported by accumulating evidence that systemic inflammation, immune competence, and nutritional status collectively influence tumor progression and treatment response. HALP integrates four routinely measured hematological parameters—hemoglobin, albumin, lymphocytes, and platelets—thereby providing a more comprehensive reflection of host–tumor interactions than single inflammatory indices such as NLR or PLR [22–26]. In our study, HALP outperformed 11 other inflammatory biomarkers across various metrics of discrimination, calibration, and time-dependent analyses, suggesting that its integrative nature offers a more stable and clinically meaningful assessment of patient status in LACC. Hemoglobin is a critical determinant of tumor oxygenation. Cancer-related anemia, often driven by chronic inflammation and cytokine-mediated suppression of erythropoiesis, promotes intratumoral hypoxia, which in turn facilitates angiogenesis, genomic instability, and resistance to radiotherapy and chemotherapy [18, 22, 24]. In cervical cancer, impaired oxygen delivery has been directly linked to poorer radiotherapy outcomes, providing a mechanistic explanation for the association between low HALP and worse OS and PFS observed in our cohort. Serum albumin serves as an indicator of both nutritional reserve and systemic inflammatory burden. Hypoalbuminemia is commonly seen in advanced malignancies and is associated with compromised immune function, reduced treatment tolerance, and unfavorable survival across various cancer types [27, 28]. While albumin alone is prognostic, its inclusion in HALP allows for simultaneous assessment of inflammatory–nutritional imbalance, which may better elucidate the significant survival differences observed among HALP-defined risk groups in our study. Lymphocytes play a central role in antitumor immune surveillance. Lymphopenia indicates immune suppression and has been linked to diminished cytotoxic T-cell activity, impaired responses to chemoradiotherapy, and accelerated disease progression [18, 29]. Conversely, platelets actively promote tumor progression by facilitating angiogenesis, shielding circulating tumor cells from immune recognition, and enhancing metastatic potential through tumor-cell–induced platelet aggregation [30, 31]. Thus, a low HALP score—characterized by decreased hemoglobin, albumin, and lymphocyte levels alongside elevated platelet counts—represents a convergence of hypoxia, malnutrition, immune dysfunction, and pro-tumor inflammation. Importantly, in our cohort, HALP stratification was independent of conventional clinicopathological factors yet strongly correlated with a wide array of inflammatory indices, reinforcing its role as an upstream integrative marker rather than merely a surrogate for tumor burden. The linear relationship between HALP and survival risk further supports a dose–response biological gradient. Collectively, these mechanisms provide a coherent rationale for why HALP consistently emerged as an independent and superior prognostic indicator in LACC, aligning with previous findings across multiple solid tumors [18, 32] and underscoring its relevance in the chemoradiotherapy context. Beyond conventional photon-based radiotherapy, emerging modalities such as high-linear energy transfer radiotherapy have demonstrated enhanced immunogenic cell death and improved immune activation, particularly when combined with immunotherapy [33]. As systemic inflammatory and immune status influences radiation response, biomarkers like HALP may help identify patients more likely to benefit from immune-modulating radiotherapy strategies.

The observed linear relationship between HALP score and survival outcomes further supports its biological stability. Unlike single-ratio markers such as NLR or PLR, which primarily reflect a single inflammatory axis and may be susceptible to transient fluctuations in neutrophil or platelet counts, HALP integrates multiple complementary physiological dimensions. By combining hemoglobin (indicative of tumor oxygenation), albumin (reflecting nutritional and inflammatory status), lymphocytes (representing adaptive immune competence), and platelets (associated with pro-tumor inflammatory activity), HALP captures a broader and more stable representation of host-tumor interactions. This multidimensional integration may mitigate the impact of short-term hematologic variability and more accurately reflect the cumulative systemic environment that influences treatment response and long-term outcomes. Consequently, the integrative nature of HALP may account for its superior discriminatory power, reduced prediction error, and sustained time-dependent performance compared to single-ratio inflammatory biomarkers.

The prognostic significance of the HALP score identified in this study aligns with a growing body of evidence across multiple solid malignancies, while also extending prior findings specifically to patients with LACC treated with CCRT. Large-scale meta-analyses have demonstrated that a low pretreatment HALP score is robustly associated with inferior survival outcomes across diverse tumor types, including gastrointestinal, lung, breast, and genitourinary cancers, underscoring its generalizability as an integrative prognostic biomarker [22, 23, 28, 32]. These pooled analyses consistently reported that HALP remained an independent predictor of OS after multivariable adjustment, supporting the biological plausibility of our findings in a cervical cancer cohort. Notably, limited data have previously addressed the prognostic role of HALP in cervical cancer. A prior study focused on operable cervical cancer demonstrated that low HALP was independently associated with recurrence risk, and that incorporating HALP into a nomogram improved predictive accuracy for recurrence-free survival [17]. A recent study reported that HALP outperformed conventional inflammatory indices, such as NLR and PLR, in predicting oncological outcomes [18]. Our results not only corroborate these observations but also expand them by comprehensively comparing 12 inflammatory and nutritional biomarkers within the same cohort. Notably, HALP consistently demonstrated superior discrimination, calibration, and time-dependent predictive stability compared to NLR, PLR, LMR, SII, SIRI, and PIV, highlighting its incremental value beyond traditional indices.

Compared to studies in other cancer types, the magnitude and stability of HALP’s prognostic effect in our cohort are comparable. For example, in lung cancer and breast cancer, HALP has been shown to outperform single inflammatory markers and to retain prognostic significance across disease stages and treatment modalities [29, 30, 34, 35]. Similarly, nomogram-based studies in nasopharyngeal carcinoma and colorectal cancer demonstrated that integrating HALP with clinicopathological variables substantially improved risk stratification beyond tumor–node–metastasis (TNM) staging alone [36, 37]. In line with these reports, our HALP-based nomograms significantly improved prognostic accuracy over both the FIGO staging system and the baseline clinical model, as evidenced by higher C-index values and significant gains in IDI and NRI. Several distinctions merit emphasis. First, unlike many prior studies that focused on a single inflammatory index or relied solely on Cox regression, our study utilized a comprehensive evaluation framework incorporating time-dependent AUCs and Brier scores, enabling a more nuanced comparison of predictive performance. Second, HALP stratification in our cohort was independent of classical clinicopathological factors, yet closely correlated with multiple inflammatory indices, suggesting that HALP captures upstream systemic host conditions rather than merely reflecting tumor burden. Third, the linear dose-response relationship between HALP and survival risk, observed in spline analyses, reinforces its biological relevance—a feature not consistently explored in earlier cervical cancer studies. Collectively, when interpreted in the context of extensive prior literature [17, 18, 22, 23, 32, 38], our findings position HALP as a particularly robust and clinically practical prognostic biomarker for LACC. By integrating immunological, inflammatory, and nutritional dimensions, HALP provides complementary information beyond FIGO staging and may facilitate refined risk stratification and personalized management in this heterogeneous patient population.

The adverse prognostic impact of HPV positivity observed in our cohort warrants mechanistic consideration. Persistent high-risk HPV infection is characterized by chronic viral antigen exposure, immune evasion mechanisms mediated by E6/E7 oncoproteins, and sustained inflammatory signaling within the tumor microenvironment [39]. While HPV-driven cervical carcinogenesis is fundamentally viral, the associated chronic immune activation and local immunosuppression may influence systemic inflammatory responses. For instance, persistent viral infection can lead to immune exhaustion, dysregulated lymphocyte function, and altered cytokine profiles, which may be reflected in peripheral blood inflammatory indices [40]. Thus, the interaction between viral oncogenesis and systemic host inflammatory status may partially explain the prognostic significance of both HPV infection and HALP observed in this study. Future investigations exploring the biological and statistical interactions between viral status and systemic inflammatory biomarkers may further elucidate this relationship.

Alternative approaches, such as X-tile or time-dependent ROC-derived optimal cutoffs, may identify statistically optimized thresholds. However, these data-driven methods may introduce overfitting or reduce reproducibility across different cohorts. In this study, median-based stratification was deliberately chosen to provide a stable and unbiased comparison across 12 inflammatory biomarkers. Importantly, spline analysis demonstrated a significant linear association between HALP and survival outcomes without evidence of nonlinearity, suggesting the absence of a biologically distinct threshold and supporting the appropriateness of median-based categorization for descriptive and clinical interpretability.

Several limitations must be acknowledged. First, this retrospective analysis conducted at two tertiary referral centers may introduce selection bias and precludes definitive causal inference. Although consecutive enrollment and predefined eligibility criteria were employed, residual confounding from unmeasured factors—such as comorbidities, socioeconomic status, or variations in supportive care—cannot be excluded. Second, systemic inflammatory and nutritional biomarkers were assessed using a single pretreatment blood sample, and dynamic changes in inflammatory status during and after CCRT were not evaluated. Since CCRT can significantly modulate immune activation, tumor hypoxia, and systemic inflammatory responses, longitudinal assessment of HALP may provide additional insights into treatment response. Fluctuations in HALP during therapy could serve as an early surrogate marker of therapeutic efficacy, reflecting improvements in tumor oxygenation, nutritional recovery, or restoration of immune competence. Future prospective studies incorporating serial HALP measurements at predefined treatment milestones may clarify whether dynamic HALP trajectories can improve early response assessment and guide adaptive treatment strategies. Third, although we comprehensively compared 12 widely used inflammatory indices, other emerging biomarkers—including circulating cytokines, immune cell subsets, molecular signatures, and radiomic features—were not evaluated. The integration of multi-omics data with blood-based markers may further refine risk stratification but requires prospective validation. Fourth, while the nomograms were internally validated using bootstrap resampling to correct for optimism, external validation in an independent cohort from a different geographical region was not performed. Therefore, the transportability of the model to other healthcare systems, ethnic populations, and institutional practice patterns remains to be confirmed. Future prospective multicenter studies, particularly those involving geographically diverse populations and standardized laboratory measurement protocols, are warranted to externally validate and recalibrate the model if necessary. Such validation is critical to ensure broader clinical applicability before implementation in routine practice. Finally, although HALP consistently demonstrated superior predictive performance, the absolute gain in discrimination was moderate. Therefore, HALP should be interpreted as a complementary biomarker rather than a substitute for established clinical staging, and its value likely lies in integration within a multidimensional prognostic framework.

## Conclusion

In conclusion, this study demonstrates that the HALP score is a robust and independent prognostic biomarker for patients with LACC treated with CCRT. Compared with other systemic inflammatory indices, the HALP score exhibited superior and more stable predictive performance. Furthermore, incorporating the HALP score into nomogram models significantly enhanced prognostic accuracy beyond conventional FIGO staging and standard clinical parameters. Due to its simplicity, low cost, and routine clinical availability, HALP-based models may represent a practical and clinically applicable tool for individualized risk stratification and outcome prediction in patients with LACC.

**AI writing statement:** No AI writing assistance was utilized in the production of this manuscript.

## Supplemental data

Supplemental data are available at the following link: https://www.bjbms.org/ojs/index.php/bjbms/article/view/13993/4149.

## Data Availability

The data that support the findings of this study are available on reasonable request from the corresponding author.
